# Orator’s Address before the Alabama Medical Association, April 17th, 1894

**Published:** 1894-06

**Authors:** W. H. Blake

**Affiliations:** Lineville, Ala.


### ORATOR'S ADDRESS BEFORE THE ALABAMA MEDICAL ASSOCIATION, APRIL 17TH, 1894.

BY W. H. BLAKE, M.D.,
LINEVILLE, ALA.

*Mr. President and Gentlemen of the Association :*

It is not without a sense of personal pride that I appear before you as the representative of the Alabama State Medical Association; nor is it without a feeling of much reluctance when I realize that the audience before me are so well prepared to criticise or appreciate what may be said.

I must address you on some subject pertaining to medicine. This, I hope, will not forfeit my claims to the attention of those of my hearers not members of the medical profession, for no subject can be more worthy the consideration of a thoughtful and intelligent people.

There are few subjects, perhaps, on which more has been written than medicine, and while we are indebted to every age for something of value, still we must admit that the literature of no one subject is possibly more burdened with error, for among medical men of the past, like those of the present time, it was too frequently the case that their ability to write was in adverse ratio to the amount written.

I wish, however, to call your attention to one redeeming characteristic of the whole of medical writers, with all their voluminous faults, their one cherished hope has been to better the condition of mankind. They had in view the common objects of being able to relieve pain and save the lives of their fellow-men. With all their bickerings and contentions over baseless theories, the champions of every pathy, had their hopes been materialized, would have made the world brighter and happier. Such a laudable aim cannot be claimed for a deal of what is far more popular literature. Another thing in behalf of these advocates of conflicting theories: their zeal stimulated research, and this, in turn, led to discoveries of the greatest importance. It was in this way that truth came out of error. A truth once demonstrated became common knowledge, on which all intelligent observers must agree, and was no longer a cause for dispute. Thus demonstrated truth became the nucleus around which is being formed the science of medicine.

Such a discovery was that of Harvey. Before his day the Masters of Medicine disputed as to the function of blood vessels. His great discovery ended these forever. These demonstrated truths were not necessarily in conflict with empirical teaching. In empiricism a statement is accepted as true because it is the opinion of some favored author; in scientific medicine proof of an assertion is demanded for its belief.

Neither are all the remedies used in rational medicine of recent discovery; opium, one of the most valuable, is one of the oldest. Near Cairo, bordering the valley of the Nile, is the ancient quarry of Djebel Tura. The rock bears an inscription in heiroglyphics, stating that it furnished building material for the kings of Egypt sixteen centuries before the Christian era. This quarry continues to be worked, for the superior qualities of the stone causes a demand for it until the present day. So with a medical remedy of merit, time only establishes it the more firmly in our estimation.

Neither do we claim that in our practical work as physicians we are entirely independent of empirical methods; but every year is enabling us to rely more and more on positive medication. The demonstration of every scientific truth that has a bearing on our practical work as physicians renders us more independent of empiricism and strengthens the claims of rational medicine to being a progressive science. These discoveries have been of various kinds. Some discovered the function of anatomical organs already known; such was that wonderfully important work of Harvey. Some discovered the new and more important application of known remedies, as did Simpson in the use of chloroform as an anesthetic. McDowell and Battey devised new and successful operations for what were before incurable maladies. Who can estimate the importance of the great discovery of Jenner? Small-pox now gives us comparatively little concern; but, according to Roberts, one epidemic alone of this disease caused the death of more than three millions of people in Mexico. Our only earthly hope of escaping similar scourges is vaccination. No living man can claim being as great a benefactor to his race as can Joseph Lister. Thousands of valuable lives are now spared to years of usefulness and enjoyment, living monuments to his fame.

Such discoveries as these mark the evolution of medicine as a science and elevate it above the plane of an empirical art.

The possession of this positive knowledge makes us feel strong in our work as physicians and surgeons, and it is on this that we base our claims to the confidence and support of an intelligent people.

It is true that our resources of this character are limited, but what would the practice of medicine be to-day without them? Surgery can justly claim a degree of scientific perfection not possessed by general medicine; but where would surgery of to-day be if deprived of three scientific truths—the discoveries, respectively, of Harvey, Long and Lister.

What the future will bring us no one can tell; but that discoveries innumerable, marvelous and fruitful await us, no one can doubt. Judging from the past, this is but a reasonable conclusion. What would the earnest student of medicine not give for a medical text-book of a century hence? There are many before me whose gray hairs tell too plainly of the many nights of anxious watching they have undergone. May it not be possible that you may yet live to see some of the greatest worries of your professional lives made matters of the past? Such was the experience of the great surgeon, Lister. What a glorious reality anesthesia must have been to him who had witnessed so much of human agony and torture under surgical operations! Ernest Renan tells us that in Lebanon there have been discovered old wills, written centuries ago, which provided that the beneficiary for a given consideration shall keep the deceased informed when the Franks shall rule over the land. If, after we are dead, the dreamings of a Koch should become a reality, it would almost quicken our cold frames in the grave if the truth could be whispered in our ears that consumption was a matter of the past. Is it expecting too much that cancer and consumption are yet to be relegated to the same limbo with the plague and scurvy? We think not; we believe not; even now we are journeying toward the Mecca of our dreams, and we look to the future as containing the true fruition of our hopes. But how is this to be accomplished? By careful, painstaking, scientific investigation; by gaining a more intimate acquaintance with nature's secret processes.

At first it was my intention to say something of the lives of several of those men whose special work has done so much to establish the claims of medicine as a science, but that remonstrance of Horace demanding brevity interposes, and I have, perhaps wisely, yielded to the hint.

I should feel derelict in my duty, however, if in this connection I should fail to make special mention of the discovery of surgical anesthesia. To the surgeon no discovery, before nor since, is of equal importance—not even the great work of Harvey itself—for the use of the ligature to arrest bleeding was known and practiced before his day. It is strange that this wonderfully important property of ether had so long remained unknown, when ether itself had been in use one hundred and ninety-eight years. It was discovered by Valerius Cordius in 1540. Chloroform had been discovered by Liebig only sixteen years when Simpson made its anesthetic properties known to the world, but then the recent discovery of the anesthetic property of ether had stimulated research in that direction, where before so little was expected, and yet so much was desired. It is not without a feeling of sectional pride that we can now confidently accord this distinguishing honor, the discovery of anesthesia, to a Southern physician, Dr. Crawford W. Long.

"In 1839, Velpeau, of Paris, described the attempts to find some agent capable of preventing pain in surgical operations as nothing less than chimerical;" and in 1846 Sir Benjamin Brodie, of London, said: "Physicians and surgeons have been looking in vain, from the days of Hypocrates down to the present time, for a means of preventing or allaying bodily pain," yet four and a half years before that statement was made surgical anesthesia under the influence of ether had become a demonstrated fact in a Georgia village in the hands of Dr. Crawford W. Long.

On the 30th day of March, 1842, Jas. M. Venable was anesthetised by Dr. Long, and underwent a surgical operation without pain. That was the beginning of a new era in the history of surgery.

About two or three years later Wells, Jackson and Morton began their experiments. "With some assistance from one another they arrived successively at the anesthetic properties of nitrous oxide gas and sulphuric ether, and Morton, with an eye single to glory and business, secured a patent on ether under the name of *Letheon.* In the Massachusetts General Hospital, October 16, 1846, four and a half years after Dr. Long's discovery, Morton administered his Letheon to a patient for Dr. J. C. Warren, and a tumor of the neck was successfully removed." Other operations soon followed, and the published reports of these cases created intense interest throughout the medical world.

"Now began the celebrated 'Ether Controversy.' Wells claimed the honor for himself, but having failed both at home and abroad to receive recognition as the discoverer, he ended his life by suicide January 14, 1848."

"In 1852 a bill was introduced in congress to purchase Morton's patent for $100,000. This was successfully opposed by Long, Jackson and the friends of Wells. Morton was greatly disappointed at not receiving the reward from congress. In New York, July 15, 1868, having fretted himself into a congestion of the brain, he drove furiously up Broadway, and leaped from his buggy near Central Park. He was taken up insensible, and carried to St. Luke's Hospital, where he died an hour later. It was not long before Jackson was committed to an asylum hopelessly insane. After some years of confinement he died August 28, 1880."

"Dr. Long continued to practice his profession in Athens, Ga., and enjoyed a good patronage. In regard to the discovery of anesthesia he had often said, 'My only wish about it is to be regarded as the benefactor of my race.' While on a visit to a patient, June 15, 1878, he was stricken with paralysis, and without regaining consciousness died the next day." He combined excellently the noblest qualities of the physician with the highest attributes of the gentleman. He claimed nothing for himself that was not his; his character was known of all men, and was above reproach; he was modest, honest and truthful to the last degree, and he "kept the covenant of his heart's true life until his days were numbered."

"Paul found in his travels many altars erected to as many gods, and in Athens another 'to the unknown God,' so the tourist can see in Hartford, Conn., a bronze statue erected to the memory of Horace Wells; in Mt. Auburn Cemetery, Boston, a marble shaft to W. T. G. Morton; a statue to Dr. Crawford W. Long is to be placed in the National Gallery of Statues in Washington; and in Boston there is a monument of white marble dedicated 'To the Discoverer of Anesthesia.' It bears no name, but to one familiar with history it recalls the sad story of hopes that were disappointed, ambitions that were defeated and lives that were lost, through their connection with the most humane and beneficent discovery of modern times."

In the cemetery at Athens, Ga., is the grave of Dr. Long. "The oaks above him and the river at his feet murmur a requiem, but no shaft of marble tells to the world that here rests one who, above all others, did most good to mankind. Humbly, as the humblest dweller in that 'city of the dead,' he sleeps whose last years were saddened with the knowledge that to others the meed of praise that should have been his was given. No thought of fame or worldly emolument entered into his heart; he asked only the honor to which he was due, and which now, too late to cheer his spirit, is showered upon his memory. An unknown and an unmarked grave is his." How tardy often is gratitude!*

I have endeavored to present the claims of medicine to the rank and dignity of a science. It is the application of scientific knowledge in our practical work as physicians. It is but one department of universal truth. Bacon has said that "no pleasure is comparable to standing on the vantage ground of truth," and no one more fully appreciates this than does the earnest student of medicine.

Now, ladies and gentlemen, I wish to call your attention to a few of the many commendable features of the Alabama State Medical Association. The most important, perhaps, of these is its function of guardianship over the public health of our State. Should epidemic or pestilence threaten our borders, Alabama is protected

*To anyone who entertains a doubt as to Dr. Long's priority in the discovery of anesthesia, I refer him to the valuable contribution on this subject by Dr. L. B. Grandy, of Atlanta, Ga., from which I have freely quoted. It appeared in the *Virginia Medical Monthly*, October, 1893.

to a degree not exceeded by any other State in the Union. Through its operation the standard of professional attainment among its membership is being made higher year by year. I do not refer to the examination of applicants who are to practice in the State, but to that diffusion of medical knowledge among the members of this Association which comes from yearly attendance at these meetings. Our membership comprises the best medical talent in the State, and the discussions which are each year conducted in our meetings are a fruitful source of improvement to those members who are not so well informed.

Another thing, membership in our Association stimulates professional pride, that element so essentially important to every professional man. This cannot be overestimated. I think every member of this audience will bear me out in the assertion, that an individual without personal or family pride, a citizen without State pride, or a professional man without professional pride, is an inferior product of his kind.

But, says some one, "this word in behalf of the Alabama State Medical Association is needless; its beneficent aims; its record of devotion to the best interests of the State; its policy of zealously avoiding entanglement in party strife—these of themselves should commend it to the indorsement and support of every patriotic citizen of Alabama."

Yes, my friend, what you say is true, but the Alabama State Medical Association has never been without opposition in the State, and its strongest opponents have always been members of the medical profession. Would you ask why this is true? The story of Simon Girty will answer the question for at least some of them:

At one time during the early settlement of our western frontier, Indian depredations became so frequent that the whites were compelled to organize themselves into a military force for self-protection. Among them was one Simon Girty, who was exceedingly anxious to be elected an officei in the new company, but failing in his efforts he deserted the whites, became a renegade, and joined the Indians in their warfare against his own people. But then we have some worthy members of the medical profession who, from indifference or lack of professional pride, hold themselves aloof from this Association. These we adjure to depart from their error and join us in our noble work.

Now, I would say to those doctors who hold themselves aloof from our Association, join us and aid us in our organized effort to better prepare ourselves to contend with the common enemies of mankind, disease and death.

There is another class of physicians whom I would mention, those who have forfeited their membership in the Association to become the employees in some bichloride of gold establishment or similar institution. Brother physician, I would beseech that for these you leaven your censure with charity. Look on these erring physicians with a certain degree of leniency. You don't know what you would do, if you needed practice as bad as they did.

But the Alabama State Medical Association is to-day more firmly rooted in public confidence than ever before. The people of Alabama are proud of it, and they have just cause to be. It is pronounced by eminent authority to be incomparably superior to any other medical organization on the American continent. This is due to that spirit of patriotism, conservatism and unselfishness that has pervaded its councils and the wisdom of its leadership.

On a bright autumn day, 480 B. C., Xerxes, king of Persia, landed on the island of Salamis; he ascended the craggy cliffs of the promontory and stood on an overhanging rock commanding a view of the Saronic Gulf. On his left, approached his own armed fleet; on his right was that of the Greeks. Xerxes stood there to watch that conflict on whose issue depended the destiny of civilization. Who was to be master of the world, Greek or Persian? The ships met; the Persians went down; civilization was saved.

This Medical Association has had its bitter fight to the death; our halls of legislation was the arena of conflict; opposition went down, the Association was saved.

One special hand has been felt in guiding its craft through many narrow breakers, until now it floats calmly on the broad and deep waters of the placid gulf. That pilot is still at his post, and may many years to come still find him there, for ocean sailing is not without its danger, but all on board feel safe when his hand is at the helm. We may fitly apply to him * those words of Horace—

" The man of firm and righteous will,
No rabble clamorous for the wrong,
No tyrant's brow whose frown may kill,
Can shake the strength that makes him strong."

Now, ladies and gentlemen, your patience just a little while longer while I say a few words as to the individual doctor. No one else sees the world as the doctor sees it; no one else knows the word as the doctor knows it; he knows of crimes that never reach the ears of a jury, and he hears confessions that never reach the ears of a priest. "The community, as a rule, view one another with a veil thrown over their moral and physical afflictions; their strong passions and feeble control; their blasted hopes and the sorrows that flow from their love and hatred; their poverty, their frailty, their crimes, and their vexations; their cruel disappointments and rude mortifications; their follies and disasters, fears, delinquencies and solicitudes; he sees the trappings of greatness and the cloak that hides deformity dropped, their infirmities and imperfections of mind and body with the veil uplifted, and the book of the heart wide open. The homeless, the betrayed, the deserted and the victims of intemperance; 'grief and joy,' hope and despair, love, guilt, debt, jealousy, shame, grief, domestic trouble, superstition, anxiety, thirst for revenge," all are known to the doctor. "He becomes the repository of all kinds of moral and physical secrets, but he closes his lips doubly tight. At one hour he visits the poor felon in his cell; the next may find him in a palace. At one hour he visits the minister of God; the next he is called to the very cesspool of vice and sin and there must listen to the dying shrieks of some victim of remorse and shame.

Such are the doctor's opportunities for seeing the world as it is, but I'll assure you that all of his ways are not ways of pleasantness and not all of his paths are peace. My hearers, do you realize the difficulties with which the doctor has to contend? "He must endure," says Dr. Cathell, "all temperatures, August suns and December blasts; drowned with the rain and choked with the

* Dr. Jerome Cochran, Montgomery, Ala.

dust, he must trudge hungry and sleepy, at noon or midnight, while others oblivious of care are resting or being refreshed with sleep." "A physician is in continual danger, and while like a wild and relentless tornado the swift, gaunt, ghastly, withering epidemic begins its work of death, no matter how great the danger, he cannot flee but in dishonor."

A few years ago the world reverberated the praises of Father Damein who himself fell a victim while administering to the spiritual needs of a leprous stricken colony. His was no greater sacrifice than was made by those physicians who voluntarily offered their services to the people of Memphis during the yellow fever epidemic of 1878. There "the pestilence was king, and panic and shuddering horror, and the black shadow of death were his ministers." None else knew so well the character of the danger they were to face, and yet physicians left their homes and voluntarily offered their services to those fever-stricken people.

These are the class of physicians who form themselves into medical associations. The highest aim of their lives is to better prepare themselves for the fearful responsibilities of their profession, their greatest reward the confidence of the people whom they serve.

In our claim for your confidence we are by no means without rivals, but who are these rivals? During that fearful scourge at Memphis, I wonder how many patent medicine men voluntarily left their homes and with their own hands offered their "never failing" remedies to those dying people? They boast of success where physicians fail—here certainly was a field for their pretended usefulness. These rivals for your confidence are of all degrees of pretention, from that arch-quack, Dr. Flower, all the way down to that peripatetic cosmopolitan, the long-haired Indian root-doctor, and their remedies are in varying favor, from an electropoise all the way down to a rabbit's foot. From the recommendations with which our religious newspapers have been burdened of late, it seems that the electropoise is specially efficacious in treating the diseases of preachers. "One of the most amazing of all wonders is that wisdom in the mysteries of the law or the doctrines of theology, acumen in the sciences, skill in the polished arts, or keenness and culture in other departments of human knowledge, scarcely increases some people's reasoning powers a jot above the ancient Egyptians in medical matters." There is a popular belief that some men are born doctors. Did you ever hear of a born chemist, a born electrician or a born engineer? A knowledge of medicine comes just like any other knowledge, by careful, painstaking investigation.

Laenec perfected the stethoscope just as Bell perfected the telephone; each studied the transmission of sound. "Professional skill," says Fothergill, "consists of a foundation of common sense with a superstructure of special education." A medical student may, by his own efforts, supply all other deficiencies, but if he lacks common sense, that element which Guizot has denominated "the genius of humanity," his failure is inevitable.

But I was speaking of the doctor's difficulties. Some one suggests that it may be attributed to the uncertainties of medicine. That medicine is not an exact science, we must admit. The same may be said of law. The decisions of to-day are liable to be reversed to-morrow. But you have an important suit at law; great interests are involved; your case is in the hands of an attorney of years of experience and recognized ability; neither you nor your lawyer can foretell the result; but what would your friends think of you if, under these circumstances, you dismiss your lawyer and put your case into the hands of some vulgar pretender, who has had neither opportunity nor inclination to acquaint himself with the known principles of law? You are caught in a storm at sea; the thunders roll and the lightnings flash, and the ship quivers and its timbers crack as it rides the surging waves, the wind blows more furiously and just yonder is the rocky reef. The old pilot stands bravely at the helm; no one else realizes the danger so well as he, but with a steady gaze on the menacing rocks he rapidly shifts the wheel to adjust the ship to the rapidly shifting winds; but what would you think of a captain who would now put at the helm an ignorant landsman who, to your own knowledge, could scarcely paddle a canoe?

Inconsistencies as glaring as these are of too frequent occurrence in the medical world. The physician has difficulties and worries and vexations. He must contend with dark nights and cold rides and poor pay, but he tastes joys as free from alloy as are ever permitted to gladden the human heart. He may miss wealth and fame, but if his life is rightly spent, no other calling can better enable him to at last exclaim: "I feel within me a peace above all earthly dignities, a clear and quiet conscience!" Such is the doctor's life.

